# Frequency, moisture content, and temperature dependent dielectric properties of potato starch related to drying with radio-frequency/microwave energy

**DOI:** 10.1038/s41598-017-09197-y

**Published:** 2017-08-24

**Authors:** Zhuozhuo Zhu, Wenchuan Guo

**Affiliations:** College of Mechanical and Electronic Engineering, Northwest A&F University, Yangling, Shaanxi 712100 China

## Abstract

To develop advanced drying methods using radio-frequency (RF) or microwave (MW) energy, dielectric properties of potato starch were determined using an open-ended coaxial-line probe and network analyzer at frequencies between 20 and 4,500 MHz, moisture contents between 15.1% and 43.1% wet basis (w.b.), and temperatures between 25 and 75 °C. The results showed that both dielectric constant (*ε*′) and loss factor (*ε*″) were dependent on frequency, moisture content, and temperature. *ε*′ decreased with increasing frequency at a given moisture content or temperature. At low moisture contents (≤25.4% w.b.) or low temperatures (≤45 °C), *ε*″ increased with increasing frequency. However, *ε*″ changed from decrease to increase with increasing frequency at high moisture contents or temperatures. At low temperatures (25–35 °C), both *ε*′ and *ε*″ increased with increasing moisture content. At low moisture contents (15.1–19.5% w.b.), they increased with increasing temperature. The change trends of *ε*′ and *ε*″ were different and dependent on temperature and moisture content at their high levels. The penetration depth (*d*_*p*_) decreased with increasing frequency. RF treatments may provide potential large-scale industrial drying application for potato starch. This research offers useful information on dielectric properties of potato starch related to drying with electromagnetic energy.

## Introduction

Starch is a major agricultural product that is consumed and/or used on a daily basis in the whole world. Its application is not only confined to making foods, but also used as raw materials in industry, such as in oil drilling, mining, and making bio-plastics, paper, and textiles^1^. Potato starch, a representative of tuber starches, is the third-place material with about 6% global starch production^2, 3^. Drying is the last process in the production of potato starch. During this period, the wet starch cake with about 40% moisture content in wet basis (w.b.) is dried to about 20% w.b. using hot air at 150 °C^4^. Although high temperature improves heating or drying efficiency, it affects rheological properties of starch granules and final quality of starch products^5, 6^. Kugimiya *et al*.^7^ studied the unrecoverable endothermic gelatinization of potato starch at high moisture contents, and found that gelatinization temperature was about 65 °C. Donovan^8^ found that when potato starch granules with moisture content lower than 60% w.b. were heated, there were two endothermic transitions. The high endothermic temperature increased with decreasing moisture content, and the low endothermic temperature was found at 66 °C. It indicates that drying below 65 °C is helpful to obtain potato starch with high quality.

Dielectric heating, which incorporates radio-frequency (RF) and microwave (MW) treatments, has been regarded as an advanced and promising technology in drying agricultural products. Some studies have indicated that RF/MW drying has advantages in improving thermal efficiency and product quality, and in shortening drying time when contrasted with conventional drying^9–13^. However, Jiang *et al*.^14^ found that drying starch using dielectric heating resulted in poor heat distribution uniformity compared to using conventional thermal energy.

Dielectric properties are main parameters that describe the interaction between electric energy and materials during dielectric heating. Study on dielectric properties of agriculture products is necessary and crucial to develop effective RF or MW drying process^15^ and to improve dielectric heating uniformity. Frequency, moisture content and temperature are main factors that influence the dielectric properties of food materials^16^. The effects of temperature and moisture content on dielectric properties of several kinds of starches were studied by Ndife *et al*. at 2450 MHz^17^. Motwani *et al*.^18^ found that the dielectric properties of starch slurries were influenced by starch concentration and gelatinization. However, few reports have been found on studying the dielectric properties of potato starch as related to dielectric drying with RF/MW energy. Moreover, little information was reported on penetration depth which influences drying uniformity. Therefore, the objectives of this study were: (1) to obtain the dielectric properties of potato starch at moisture contents between 15.1% and 43.1% w.b. and at temperatures between 25 and 75 °C over a frequency range from 20 to 4,500 MHz, (2) to analyze the relationship between dielectric properties and penetration depth with above-mentioned factors, and (3) to provide information on designing drying process and on deciding treatment sample thickness for drying potato starch with RF/MW energy.

## Results and Discussions

### Effect of frequency on dielectric properties

**Figure 1:**
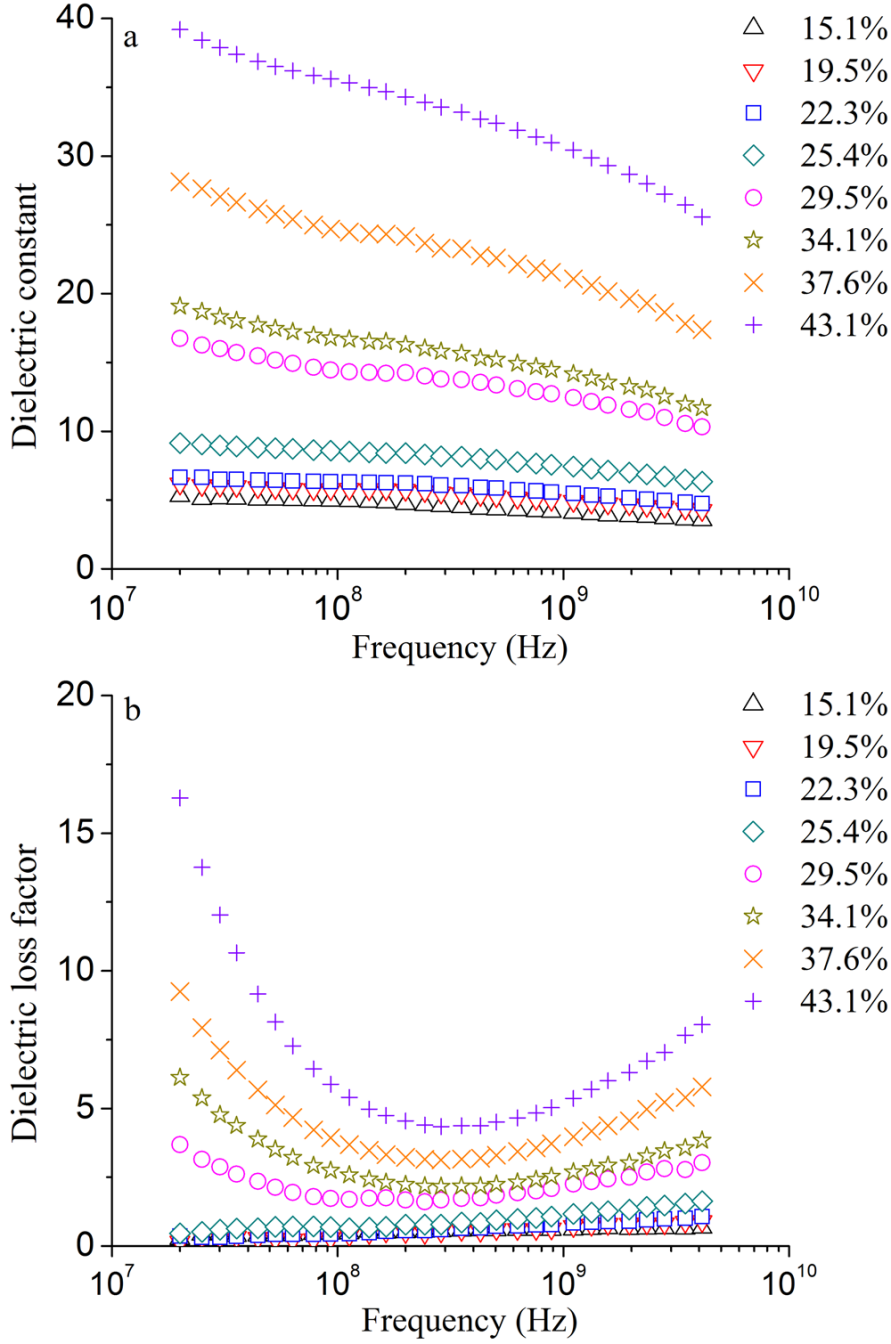
Obtained mean values of *ε*′ (**a**) and *ε*″ (**b**) of potato starch samples with moisture contents of 15.1% (Δ), 19.5% (∇), 22.3% (□), 25.4% (◇), 29.5% (○), 34.1% (☆), 37.6% (×), and 43.1% (+) w.b. at 35 °C and over the frequency range of 20–4,500 MHz.

Obtained mean values of *ε*′ and *ε*″ of potato starch samples with different moisture contents at 35 °C and over the frequency range of 20–4,500 MHz are shown in Fig. 1. It was found that both *ε*′ and *ε*″ were influenced by frequency. *ε*′ decreased with increasing frequency at all moisture contents (Fig. 1a). At higher moisture contents, the effect of frequency on *ε*′ was a little clearer than that at lower moisture contents. For example, *ε*′ of potato starch with 43.1% w.b. decreased from 39.19 to 25.07 when the frequency increased from 20 to 4,500 MHz, decreasing by 36.03%. However, it decreased from 5.28 to 3.50, decreased by 33.71%, when the moisture content was 15.1% w.b. (Fig. 1a). Same change trend was also noted on the potato starch samples at other temperatures.

The dielectric constant of pure polar materials could be described using a mathematical formulation developed by Debye^19^, which is

1 $$\varepsilon ^{\prime} ={\varepsilon }_{\infty }+\frac{{\varepsilon }_{{\rm{s}}}-{\varepsilon }_{\infty }}{1+{(2\pi f)}^{2}{\tau }^{{\rm{2}}}}$$

where *ε*_*s*_ represents the static dielectric constant, i.e., the dielectric constant value at zero frequency, *ε*_∞_ represents the dielectric constant at frequencies so high that molecular orientation does not have time to contribute to the polarization, and *τ* is the relaxation time in second.

Equation (1) indicates that the value of *ε*′ is determined by *ε*_*s*_, *ε*_∞_, *f* and *τ*. At a given temperature, *ε*_*s*_, *ε*_∞_ and *τ* are almost constants. Therefore, *ε*′ decreased with the increase of frequency. Similar trend was also observed on other agriculture products and foods, such as wheat flour^20^ and ground almond shells^21^ over the frequencies from 10 to 1800 MHz, and cheese between 300 and 3000 MHz^22^.

It can be found that for the potato starch, *ε*′ decreased almost linearly with increasing frequency in the logarithmic scale (Fig. 1a). Their linear relationship could be described by following equation:

2 $$\varepsilon ^{\prime} =a\,{\mathrm{log}}_{10}f+b$$

where *a* and *b* are regression constants. The values of regression constants and the linear coefficients of determination (*R* ^2^) of Equation (2) at investigated moisture contents and some temperatures are given in Table 1. Table 1 tells that *R* ^2^ are higher than 0.9 with a few of exceptions, indicating that Equation (2) could be used to express the relationship between frequency and dielectric constant. The almost negative linear relationship between *ε*′ and log_10_ *f* was also found by Everard *et al*.^22^ on cheese at 300–3000 MHz.

**Table 1:** The regression constants and the linear coefficients of determination of Equation (2) at investigated moisture contents and several temperatures.

Moisture content, % w.b.	35 °C			55 °C			75 °C		
	a	b	*R* ^2^	a	b	*R* ^2^	a	b	*R* ^*2*^
15.1	−0.7570	10.898	0.9749	−0.8537	13.753	0.8130	−3.3212	52.426	0.9434
19.5	−0.8021	12.163	0.9536	−2.2263	32.863	0.9243	−2.8793	46.531	0.9130
22.3	−0.8109	12.775	0.9409	−3.4136	49.567	0.9697	−2.8690	48.598	0.9115
25.4	−1.1524	17.762	0.9596	−3.6171	52.517	0.9770	−2.0735	37.625	0.7889
29.5	−2.4259	34.140	0.9762	−3.1276	49.561	0.9280	−2.1102	38.682	0.7715
34.1	−2.8656	39.846	0.9833	−2.1554	34.563	0.9227	−1.2816	26.939	0.5797
37.6	−4.1366	58.088	0.9797	−2.5069	39.817	0.9344	−2.2494	41.189	0.8597
43.1	−5.3093	77.979	0.9794	−4.3555	68.450	0.9812	−2.8768	54.575	0.9067

Figure 1b shows that *ε*″ was affected not only by frequency, but also by moisture content. When the moisture content was lower than and equal to 25.4% w.b., *ε*″ almost increased linearly with increasing frequency in logarithmic scale. However, *ε*″ changed from decrease to increase with increasing frequency above 25.4% w.b. The minimum *ε*″ was found at about 300 MHz.

Dielectric losses are caused by Maxwell-Wagner polarization, ionic conduction, dipole, electronic, and atomic mechanisms^23^. For the food materials and agricultural products with high moisture content, ionic conduction and dipole relaxation are main dielectric loss mechanisms^24^. Ionic conduction contributes mainly on dielectric loss at radio frequency range below 300 MHz, and dipole polarization plays a major role at microwave frequencies^25, 26^. In this study, for the potato starch samples with moisture content higher than 25.4% w.b., ionic conduction was dominated loss mechanism below about 200 MHz, and dipole polarization was main loss mechanism above 200 MHz.

The monotonically increased *ε*″ with frequency at low moisture content was also found on macadamia nut kernels with moisture content of 3% over the frequency range of 10–1,500 MHz^27^. The change trend of *ε*″ which decreased firstly then increased later with increasing frequency has been reported on many agricultural products and food materials at high moisture content, such as on goat’s milk^28^, corn flour^29^, chicken meat^30^, and edible oils^31^.

**Figure 2:**
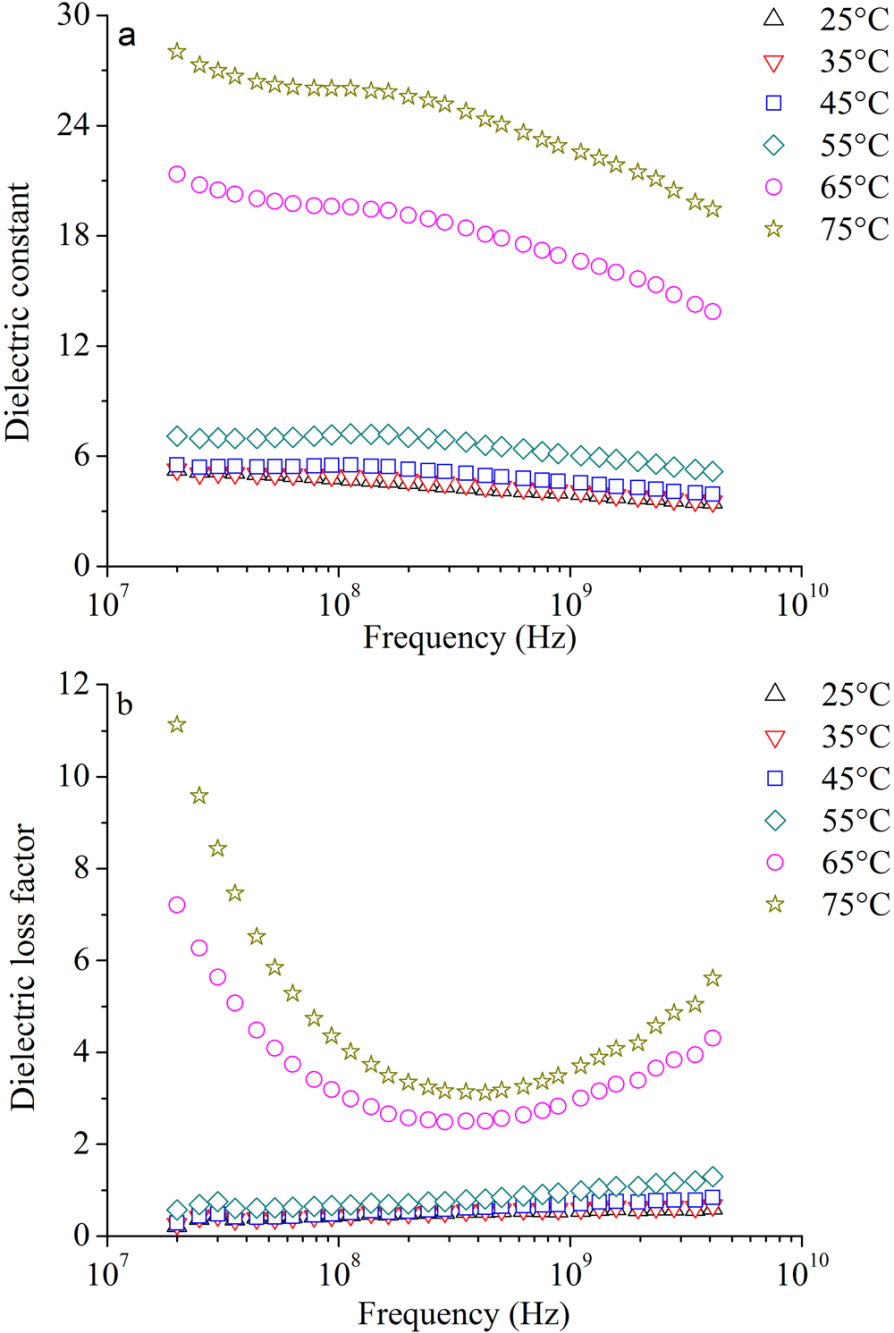
Obtained mean values of *ε*′ (**a**) and *ε*″ (**b**) of potato starch samples with the moisture content of 15.1% w.b. at 25 (Δ), 35 (▽), 45 (□), 55(◇), 65 (○), and 75 °C (☆) and over the frequency range of 20–4,500 MHz.

Obtained mean values of *ε*′ (a) and *ε*″ (b) of potato starch sample with moisture content of 15.1% w.b. at investigated temperatures and over the frequency range of 20–4,500 MHz are shown in Fig. 2. At every investigated temperature, *ε*′ decreased almost linearly with increasing frequency (Fig. 2a). It means that the temperature had no effect on the change trend of *ε*′ with frequency. However, the decrease was more obvious at 65 °C and 75 °C than at other temperatures. Equation (2) also could be used to describe the frequency dependence of *ε*′ at investigated temperatures. When the temperature was lower than and equal to 55 °C, *ε*″ increased slightly and almost linearly with increasing frequency (Fig. 2b). At 65 °C and 75 °C, *ε*″ decreased with increasing frequency until about 400 MHz, and then increased with the increase of frequency.

### Effect of moisture content and water activity on dielectric properties

**Figure 3:**
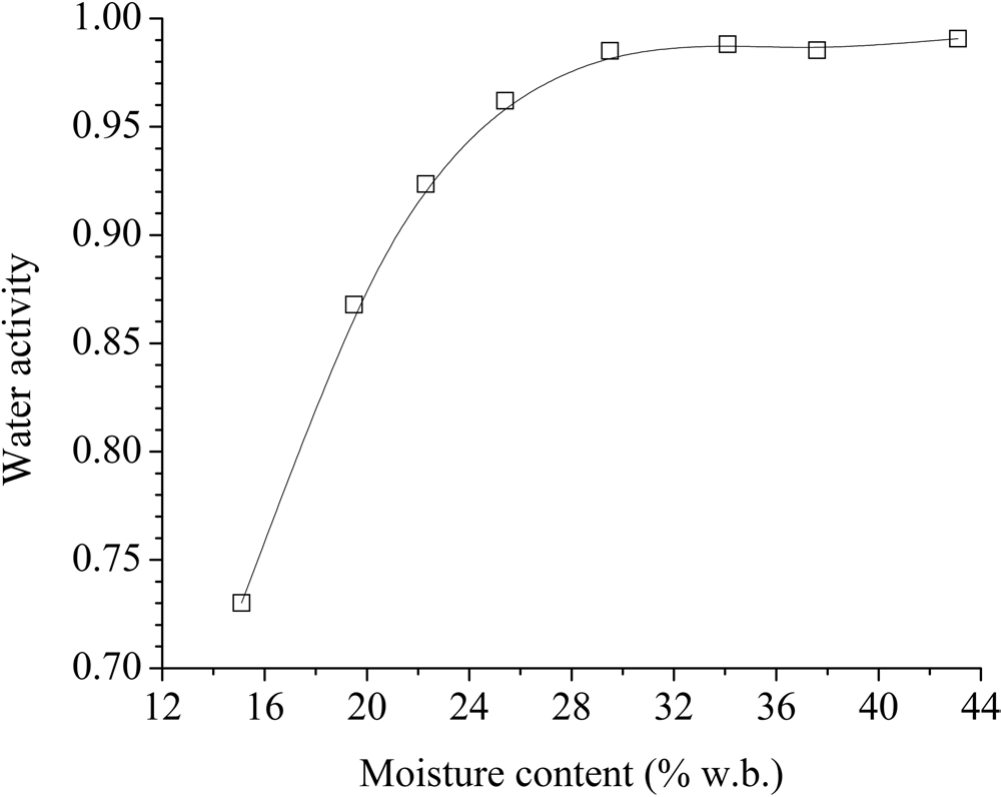
Relationship between water activity and moisture content of potato starch samples at 25 °C.

Figure 3 shows the relationship between water activity and moisture content (moisture sorption isotherm) of potato starch samples at 25 °C. It indicates that the water activity increased greatly with an increase of moisture content when the moisture content was less than 25.4% w.b. Then the water activity increased a little from 0.95, until kept at a constant value of 0.99 when the moisture content was higher than 25.4% w.b.

**Figure 4:**
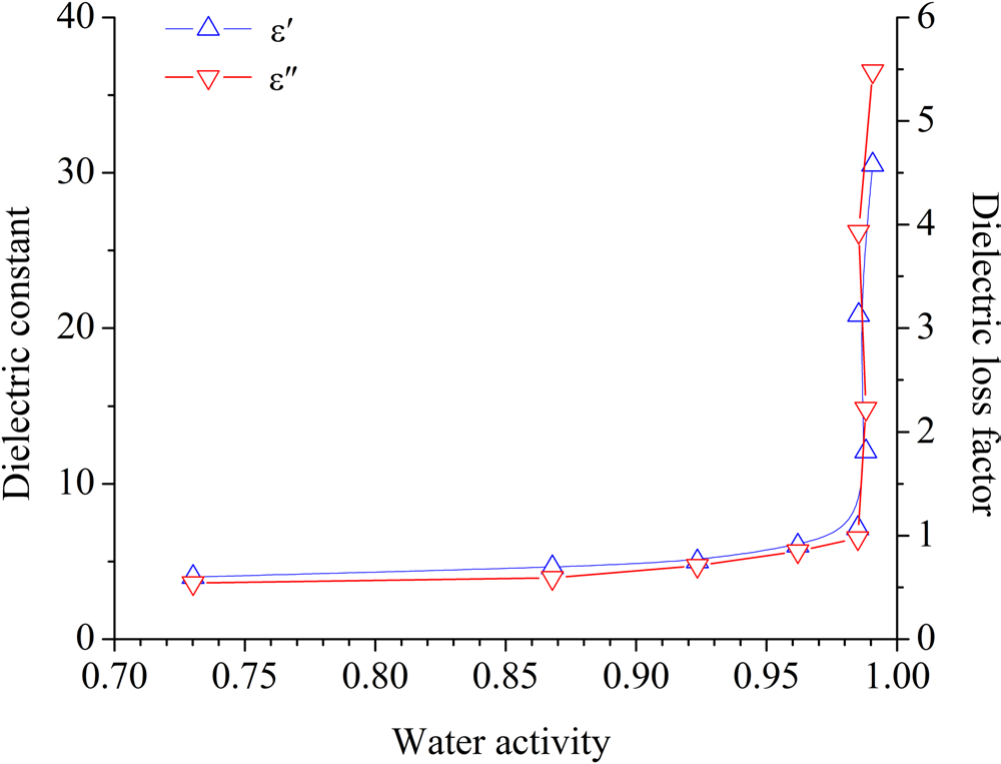
Obtained mean values of *ε*′ (Δ) and *ε*″ (∇) of potato starch samples as the function of water activity at 25 °C and 915 MHz.

Figure 4 shows the influence of water activity on dielectric properties of potato starch samples at 25 °C and 915 MHz. The results show that both *ε*′ and *ε*″ varied hyperbolically with water activity. They increased slowly at water activities from 0.73 to 0.97, and then increased very sharply above 0.97. The influence of water activity on permittivities agrees with the dielectric behavior observed on apples^32^ and potatoes^33^. This result tells that the dielectric loss mechanism would be improved notably if the water activity is higher than a value, for example 0.97 here. The sharply increased permittivities are related to availability of mobile water.

**Figure 5:**
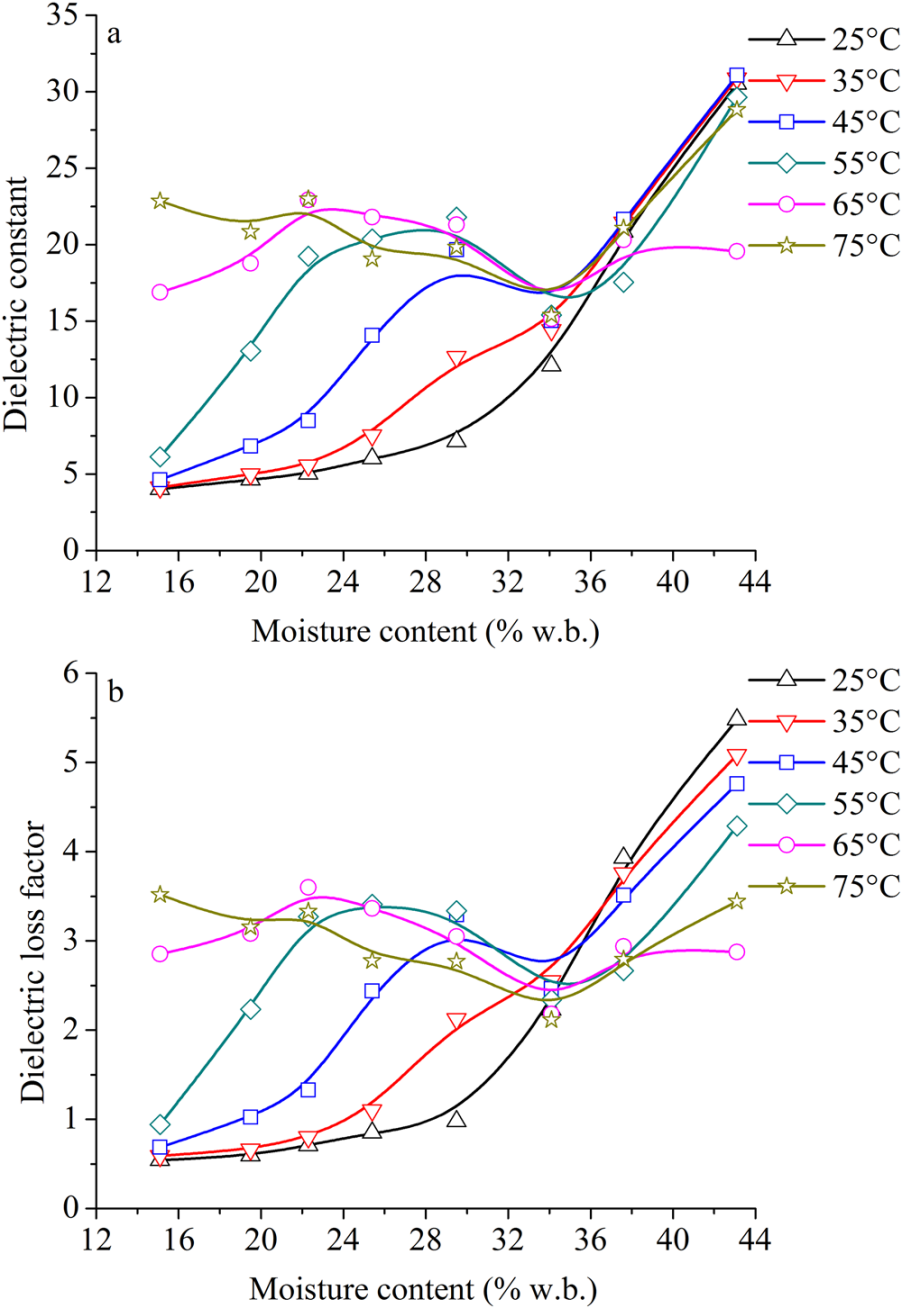
Obtained mean values of *ε*′ (**a**) and *ε*″ (**b**) of potato starch samples as a function of moisture content range from 15.1% to 43.1% w.b. at 25 (Δ), 35 (∇), 45 (□), 55(◇), 65 (○), and 75 °C (☆) and 915 MHz.

Figure 5 shows the influence of moisture content from 15.1% to 43.1% w.b. on *ε*′ and *ε*″ of potato starch samples at indicated temperatures and 915 MHz. Obviously, the dielectric properties are moisture content dependent. However, the change trend of permittivities with moisture content was influenced by temperature. Free water and bound water are two water types in agricultural products and foods. The former is the main water type in the materials with high moisture content, while the latter is the main water type at low moisture content. The effect of free water on dielectric properties is much greater than that of bound water.

At 25 and 35 °C, both *ε*′ and *ε*″ increased slowly with moisture content below 25.4% w.b., then increased greatly above 25.4% w.b. This means that the main factor affecting dielectric properties was bound water and free water at low and high moisture contents, respectively. The increasing permittivities with moisture content have been reported on chickpea flour^34^, legume samples^35^, and almond kernels^36^.

It was noted that when the moisture content was lower than 34.1% w.b., both *ε*′ and *ε*″ increased with an increase of moisture content firstly, and then decreased at 45, 55 and 65 °C. However, they decreased monotonically with increasing moisture content at 75 °C. When the moisture was higher than 34.1% w.b., both *ε*′ and *ε*″ increased greatly at 45, 55, 65, and 75 °C. This indicates that free water became dominant factor on dielectric behavior of potato starch. The permittivity values at some low moisture contents were higher than at some high moisture contents at a given temperature. This phenomenon was also found by Motwani *et al*.^18^. It is likely that with an increase in the amount of starch in the system, the dielectric relaxation effects due to bound water in the molecular chains in the starch polymer start to interact with and possibly modify the dielectric influence of free water in the system^18^.

### Effect of temperature on dielectric properties

**Figure 6:**
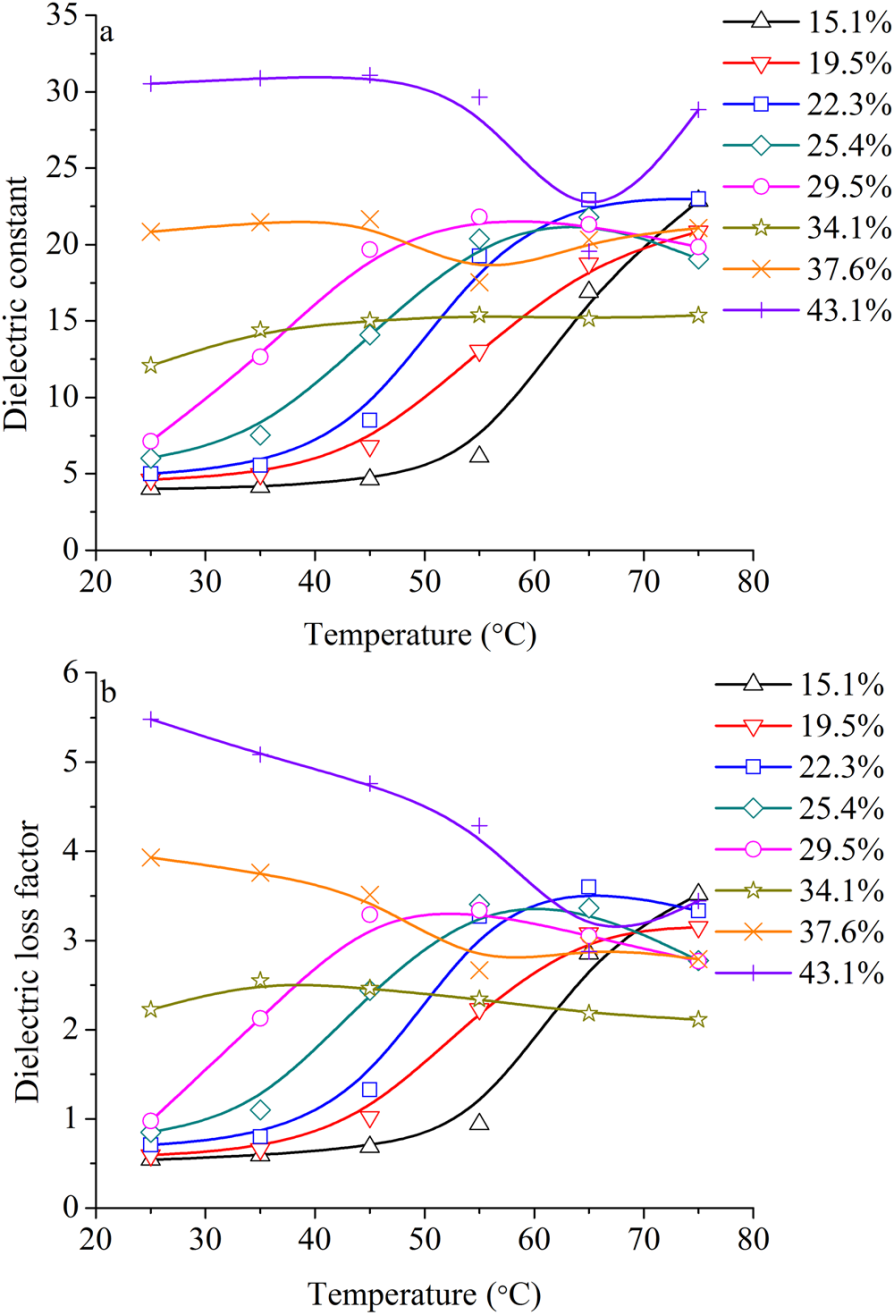
Obtained mean values of *ε*′ (**a**) and *ε*″ (**b**) of potato starch samples as a function of temperature range from 25 to 75 °C at 15.1% (Δ), 19.5% (∇), 22.3% (□), 25.4% (◇), 29.5% (○), 34.1% (☆), 37.6% (×), and 43.1% (+) w.b. and 915 MHz.

The effect of temperature between 25 and 75 °C on permittivities of potato starch samples at indicated moisture contents and 915 MHz is shown in Fig. 6. Obviously, the change trends of *ε*′ and *ε*″ with temperature were affected by moisture content.

When the moisture content was 15.1% and 19.5% w.b., both *ε*′ and *ε*″ increased with increasing temperature, especially above 45 °C. This might lie in increased dominant bound water relaxation by temperature^16^. The increased permittivities with increasing temperature at low moisture content were also reported on ground hazelnuts^37^, beans^38^, and peanut kernels^39^.

When the moisture content was 22.3%, 25.4%, and 29.5% w.b., *ε*′ and *ε*″ changed from increase to decrease and their maximum values were at 65 °C, 60 °C, and 55 °C, respectively. When the moisture content was 34.1% w.b., both *ε*′ and *ε*″ changed a little, even kept almost constant over the whole temperature. Same phenomenon was also noted by Miller *et al*.^40^ when they heated starch-water mixture at the starch-water ratio of 1:1 and 1:2 on a dry weight basis at 30–90 °C.

When the moisture contents were 37.6% and 43.1%, *ε*′ almost kept constant below 45 °C. Then it changed from decrease to increase with increasing temperature. Over the whole investigated temperature range, *ε*″ decreased firstly then increased a little later. The negative effect of temperature on dielectric values was also reported on starch solution and granular starches^17^.

Increased permittivity might lie in increased ionic conductivity and dipole polarization of free water by temperature. However, starch has unique property of gelatinization^41^. When the temperature was higher than 50 °C, reversible granule swelling occurs in starch granule^42^ and gelatinization happens in starch, which results in the decrease of water mobility, then reduced dielectric properties^43, 44^. Therefore, the decrease of *ε*′ and *ε*″ might be associated with starch granules swelling and starch gelatinization. In this study, the gelatinization temperature of the potato starch samples was 55 °C for 37.6% w.b. and 65 °C for 43.1% w.b.

### Penetration depth

**Figure 7:**
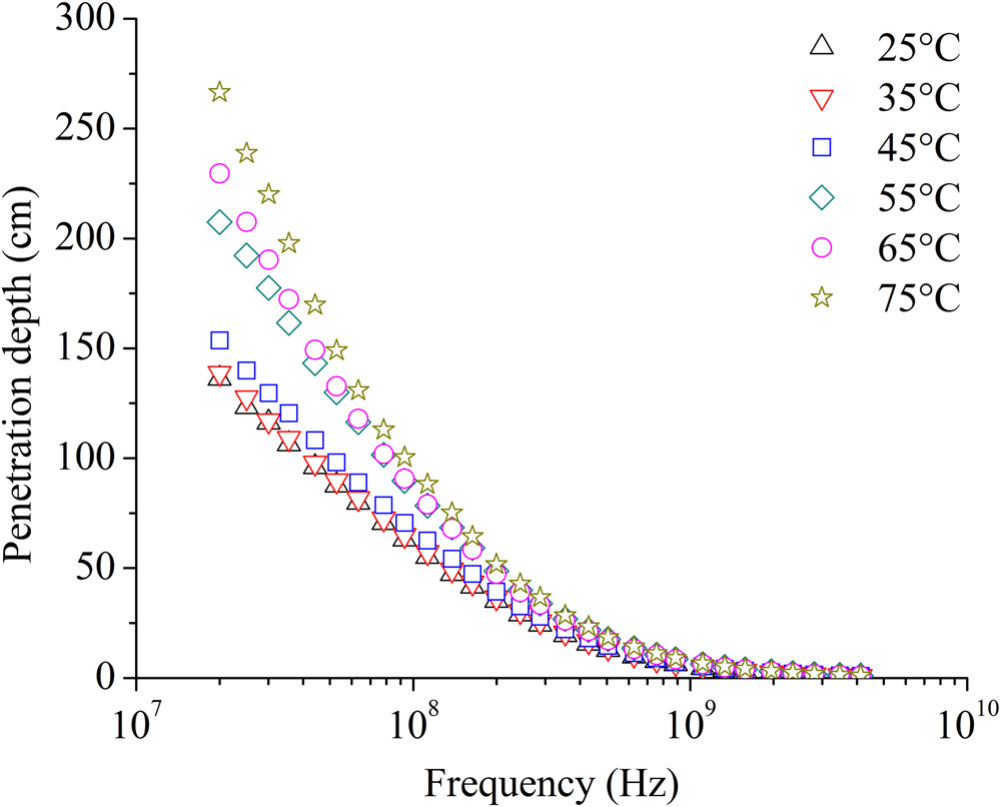
Calculated penetration depth of potato starch samples with the moisture content of 37.6% w.b. at 25 (Δ), 35 (∇), 45 (□), 55(◇), 65 (○), and 75 °C (☆) and over the frequency range of 20–4,500 MHz.

Calculated *d*_*p*_ using obtained permittivities of potato starch samples with the moisture content of 37.6% w.b. at investigated temperatures and over the frequency range of 20–4,500 MHz is shown in Fig. 7. It presents that *d*_*p*_ decreased with increasing frequency. Similar results were also found at other moisture contents. At 27.12, 40.68, 915, and 2450 MHz, *d*_*p*_ changed from 120.94 to 232.85, from 100.04 to 180.41, from 6.07 to 8.57, and from 1.62 to 2.21 cm, respectively, when the temperature increased from 25 to 75 °C. At a given temperature, the values of *d*_*p*_ at 27.12 MHz were much bigger than at other frequencies, i.e., 40.68, 915, and 2,450 MHz. The decreased *d*_*p*_ with increasing frequency was also reported on peanut kernels^39^, fruits^45^, and legume flour^46^.

**Figure 8:**
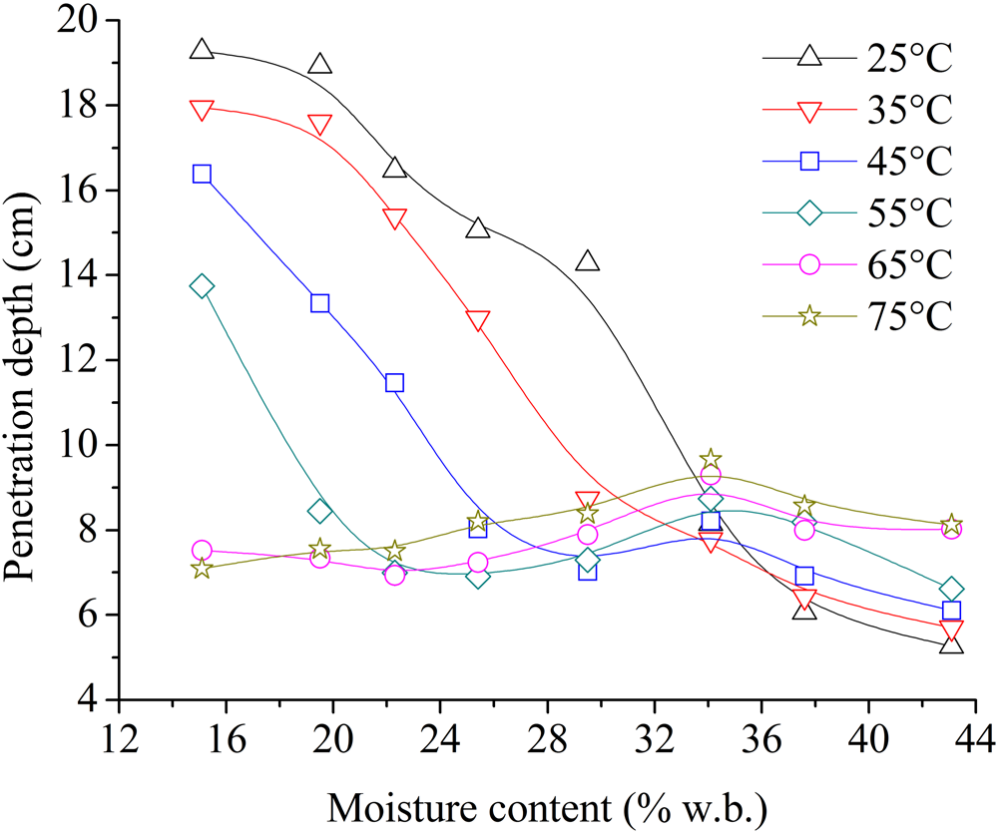
Moisture content dependent penetration depth at temperatures of 25 (Δ), 35 (∇), 45 (□), 55(◇), 65 (○), and 75 °C (☆) and 915 MHz.

Figure 8 shows the moisture content dependent *d*_*p*_ at investigated temperatures and 915 MHz. The results show that at 25 and 35 °C, *d*_*p*_ decreased with increasing moisture content over the whole moisture range. This trend agreed with the results reported for bread^47^ and red delicious apples^48^. At 45 and 55 °C, *d*_*p*_ decreased quickly with the increase of moisture content in low moisture content range (<25.4% w.b.), then changed slightly with moisture content above 25.4% w.b. Analysis of variance (ANOVA) results showed that there was no significant difference (*p* > 0.05) between the values of *d*_*p*_ at high moisture contents (≥25.4% w.b.). At 65 and 75 °C, when the moisture content was lower than 34.1%, *d*_*p*_ increased slightly with increasing moisture content, and then decreased a little. There was no significant difference (*p* > 0.05) between the values of *d*_*p*_ at all investigated moisture contents at 65 and 75 °C.

**Figure 9:**
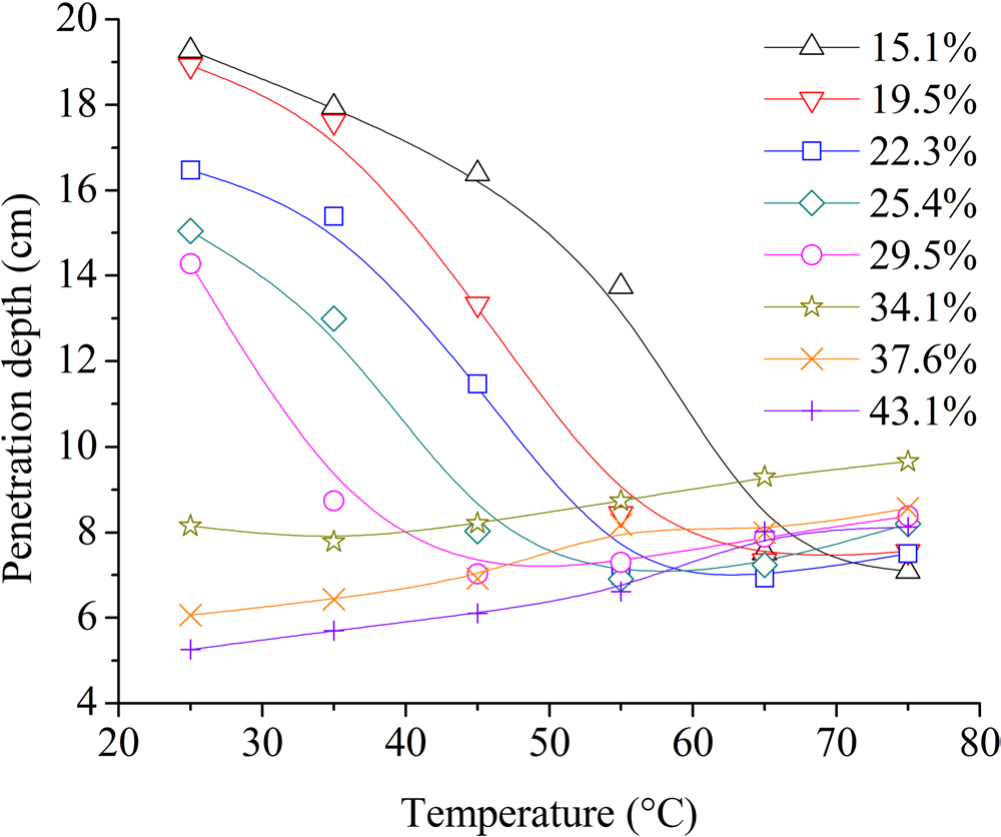
Temperature dependent penetration depth at moisture contents of 15.1% (Δ), 19.5% (∇), 22.3% (□), 25.4% (◇), 29.5% (○), 34.1% (☆), 37.6% (×), and 43.1% (+) w.b. and 915 MHz.

Figure 9 shows the temperature dependent *d*_*p*_ at investigated moisture contents and 915 MHz. When the moisture content was between 15.1% and 29.5%, *d*_*p*_ decreased with increasing temperature firstly, then changed small or even kept constant. Similar results have been reported for mango puree^49^, chestnut^50^, and rapeseed^51^. When the moisture content was higher than and equal to 34.1% w.b., *d*_*p*_ increased slightly and linearly with increasing temperature. ANOVA results showed that there was no significant difference (*p* > 0.05) between the values of *d*_*p*_ at two adjacent temperatures. The increased *d*_*p*_ with increasing temperature was also noted on some cereals, such as corn, sorghum and wheat at 915 MHz^52^, and garlic at 2450 MHz^53^.

Since *d*_*p*_ of potato starch was influenced by frequency, moisture content and temperature greatly, choosing a appropriate thickness during drying using RF/MW energy is very important. Schiffmann^54^ suggested that for uniform treatment with RF/MW energy, the largest thickness of materials should be two or three times the penetration depth. In this study, the smallest *d*_*p*_ at 27.12, 40.68, 915, and 2450 MHz at all investigated moisture contents and temperatures were 77.10, 68.02, 5.26, and 1.41 cm, respectively. If the largest thickness of potato starch was calculated as two times penetration depth, the thickness of potato starch should be smaller than 154.20, 136.04, 10.52, and 2.82 cm when the frequency of electric field was 27.12, 40.68, 915, and 2450 MHz, respectively. Large penetration depths at RF frequencies mean better heating uniformity than at MW frequencies in potato starch. Therefore, RF frequency may provide potential large-scale industrial drying applications for potato starch. It should be mentioned that even if the thickness of treated materials was smaller than the largest thickness, non-uniformity still exists during heating. Some parts might absorb more energy, and other parts might absorb less energy, which cause different temperature and different moisture content. In industry applications, agitating, shaking, moving and other methods can be used to improve heating uniformity and to maintain the temperature of potato starch below 66 °C.

## Methods

### Potato starch

Edible potato starch, produced by Beijing Dezhongjiaxin Economic and Trade Co., Ltd. (Beijing, China), was purchased from a local supermarket in Yangling, Shaanxi, China. Its main compositions in mass were 76.1% carbohydrate, 7.2% protein, 0.5% fat, and 15.1% moisture content in wet basis (w.b.). The carbohydrate, protein, and fat contents were measured according to the methods of CFR 101.9, AOAC 955.04, and AOAC 992.06, respectively. The moisture content was measured according to the standard of AOAC 925.10 with some modification.

### Sample preparation

To obtain samples at different moisture levels from original moisture content (15.1% w.b.) to about 45% w.b. with about 4% interval, 200 g original potato starch of each lot was sprayed with predetermined mass of distilled water. The samples were conditioned about 3–6 days at 4 °C in sealed polyethylene bags before they were warmed up to ambient temperature for moisture content determination and dielectric properties measurement. To ensure the moisture was distributed uniformly throughout the samples, the bags were shaken 3–4 times each day. Finally, eight moisture contents, 14.8 ± 0.1, 19.5 ± 0.1, 22.3 ± 0.2, 25.4 ± 0.1, 29.5 ± 0.2, 34.1 ± 0.2, 37.6 ± 0.3, and 43.1 ± 0.4% w.b. were obtained in this study.

### Determinations on moisture content and water activity

According to the standard of AOAC 925.1, moisture content was determined by drying triplicate 2–3 g potato starch samples on aluminum moisture dishes at 130 °C in a forced-air oven (WG-71, Tianjin Taisite Instrument Co., Ltd., Tianjin, China) until the weight kept constant. The samples were cooled in a desiccator with CaSO_4_ before reweighing to determine water loss. Moisture content was calculated from the initial and final weights of the samples. The mean value of moisture content of each sample was obtained from three replicates.

Several studies have shown that there is relationship between dielectric properties and water activity^55, 56^. Therefore, the water activity of each sample was measured at room temperature (25 °C) using an AquaLab water activity meter (4TE, Decagon Devices, Inc., Pullman, WA, USA) with an accuracy of ±0.003 a_w_. Before water activity measurement, the meter was calibrated using a standard salt solution. Then about 5 g of potato starch was placed in the measurement chamber, and the water activity was measured. Triplicate was conducted for each moisture level, and the mean value was presented.

### Dielectric properties measurement

The dielectric properties measurement system consisted of a vector network analyzer (E5071C, Agilent Technologies, Penang, Malaysia), an open-ended coaxial probe (85070E), dielectric probe kit software (85070), a constant temperature water bath (DK-98-1, Tianjin Taisite Instrument Co., Ltd., Tianjin, China), a temperature-controlled stainless steel cylindrical sample holder (23 mm in diameter and 25 mm in height), a type-T thermocouple temperature meter, a hydraulic platform, and a computer. The detailed information on the system and the calibration procedure was described earlier by Guo *et al*.^57^. In this study, the frequency range of the network analyzer used to obtain the dielectric properties was set from 20 to 4,500 MHz. The dielectric properties were measured at 201 frequencies on a logarithmic scale. The frequency range includes the specific frequencies of 27.12, 40.68, 915 and 2450 MHz, which are generally applied in ISM (Industrial, Scientific and Medical) applications.

At each moisture level, 20 g potato starch was placed in the temperature-controlled stainless steel cylindrical sample holder, which was placed in the constant temperature water bath. The temperature meter was inserted into the center of potato starch to monitor sample temperature. The water bath was put on a hydraulic platform, which was lifted to ensure the potato starch sample contact tightly with the coaxial probe. The temperature of samples was set from 25 to 75 °C with an interval of 10 °C in sequence. After the temperature of samples reached the set value, dielectric properties measurements over the frequency of 20–4,500 MHz were conducted for three times in 2 min. During dielectric properties measurements, a rubber slice with a hole in the center was used to cover the potato starch to prevent moisture loss. After the dielectric properties data were obtained, the sample height was measured, followed by measuring the sample weight. All measurements were carried out in triplicate. Mean values of nine measurements in triplicate were calculated and used for analysis. The results showed that the weight loss during dielectric properties measurement was less than 5%, and the sample density was between 0.63 g/cm^3^ and 0.67 g/cm^3^. Since the sample density change was small, the influence of density on dielectric properties was not considered in this study.

### Penetration depth

Penetration depth (*d*_*p*_, m) is defined as the depth to which the power density reduces to 1/*e* (*e* is equal to 2.71828) of its value at the surface. The depth is a key factor that influences heating uniformity and in designing the thickness of sample during RF/MW heating. When the nonmagnetic materials are under the condition of plane wave incidence, the penetration depth can be calculated as^23^

3 $${d}_{p}=\frac{c}{2\pi f\sqrt{{\rm{2}}\varepsilon ^{\prime} [\sqrt{{\rm{1}}+(\frac{\varepsilon ^{\prime\prime} }{\varepsilon ^{\prime} })}-1]}}$$

where *c* is the speed of light in free space, 3 × 10^8^ m/s, *f* is the frequency of electromagnetic wave in Hz. After obtaining the dielectric properties, the penetration depths of potato starch samples were calculated at eight moisture contents, six temperatures, and 201 frequencies.

### Data availability

The data generated and analyzed during the current study are available from the corresponding author on reasonable request.

## Conclusions

Permittivities of potato starch were measured between 20 and 4,500 MHz at moisture contents from 15.1% to 43.1% w.b. and temperatures between 25 and 75 °C. The study indicates that the permittivities of potato starch were influenced by frequency, moisture content, and temperature. *ε*′ decreased with increasing frequency at any given temperature and moisture content. The change trend of *ε*″ with frequency was influenced by temperature and moisture content. *ε*″ increased with frequency at either low temperatures or low moisture contents. However, at high temperatures or moisture contents, *ε*″ decreased firstly with increasing frequency, then increased later. Both *ε*′ and *ε*″ increased with increasing moisture content and temperature at low temperatures (25 °C–35 °C) and low moisture contents (15.1–19.5% w.b.). At high moisture contents and temperatures, the effects of moisture content and temperature on dielectric properties were complex. The penetration depth decreased with increasing frequency over the whole frequency range. The moisture content and temperature had little effect on penetration depth at their high levels, but had significant influence at their low levels.

The new frequency, temperature and moisture dependence permittivity data provide crucial information that can be useful in understanding the behavior of potato starch exposed to radio frequency and microwave dielectric heating. They also provide background information that is helpful to design drying process and apparatus using electromagnetic energy.

## References

[1] Ellis RP (1998). Starch production and industrial use. Journal of the Science of Food & Agriculture.

[2] Fornal J (2012). Influence of some chemical modifications on the characteristics of potato starch powders. Journal of Food Engineering.

[3] Haase NU, Plate J (1996). Properties of potato starch in relation to varieties and environmental factors. Starch-Starke.

[4] Grommers, H. E. & van der Krogt, D. A. Chapter 11 - potato starch: production, modifications and uses in *Starch (Third Edition)* (eds BeMiller, J. & Whistler, R.) 511–539 (Academic Press, 2009).

[5] Bergthaller W, Witt W, Goldau H-P (1999). Potato starch technology. Starch-Starke.

[6] Singh, N., Kaur, A., Shevkani, K. & Ezekiel, R. Potato: production, composition and starch processing in *Advances in food science and nutrition* (eds Visakh, P. M., Iturriaga, L. B. & Ribotta, P. D.) 23–48 (John Wiley & Sons, Inc., 2013).

[7] Kugimiya M, Donovan JW, Wong RY (1980). Phase transitions of amylose-lipid complexes in starches: a calorimetric study. Starch-Starke.

[8] Donovan JW (1979). Phase transitions of starch-water system. Biopolymers.

[9] Albanese D, Cinquanta L, Cuccurullo G, Di Matteo M (2013). Effects of microwave and hot-air drying methods on colour, β-carotene and radical scavenging activity of apricots. International Journal of Food Science & Technology.

[10] Leone A, Tamborrino A, Romaniello R, Zagaria R, Sabella E (2014). Specification and implementation of a continuous microwave-assisted system for paste malaxation in an olive oil extraction plant. Biosystems Engineering.

[11] Wang Y (2014). Developing hot air-assisted radio frequency drying for in-shell macadamia nuts. Food & Bioprocess Technology.

[12] da Silva AC (2016). Microwave drying and disinfestation of Brazil nut seeds. Food Control.

[13] Vadivambal R, Jayas DS (2007). Changes in quality of microwave-treated agricultural products - a review. Biosystems Engineering.

[14] Jiang H, Zhang M, Fang Z, Mujumdar AS, Xu B (2016). Effect of different dielectric drying methods on the physic-chemical properties of a starch-water model system. Food Hydrocolloids.

[15] Sosa-Morales ME, Valerio-Junco L, Lopez-Malo A, Garcia HS (2010). Dielectric properties of foods: reported data in the 21st century and their potential applications. LWT-Food Science and Technology.

[16] Calay RK, Newborough M, Probert D, Calay PS (1995). Predictive equations for the dielectric properties of foods. International Journal of Food Science & Technology.

[17] Ndife MK, Sumnu G, Bayindirli L (1998). Dielectric properties of six different species of starch at 2450 MHz. Food Research International.

[18] Motwani T, Seetharaman K, Anantheswaran RC (2007). Dielectric properties of starch slurries as influenced by starch concentration and gelatinization. Carbohydrate Polymers.

[19] Nelson, S. O. & Datta, A. K. Dielectric properties of food materials and electric field interactions in *Handbook of microwave technology for food applications* (eds Datta, A. K. & Anantheswaran R. C.) 70–75 (Marcel Dekker Inc., 2001).

[20] Nelson SO, Trabelsi S (2006). Dielectric spectroscopy of wheat from 10 MHz to 1.8 GHz. Measurement Science and Technology.

[21] Gao M, Tang J, Johnson JA, Wang S (2012). Dielectric properties of ground almond shells in the development of radio frequency and microwave pasteurization. Journal of Food Engineering.

[22] Everard CD, Fagan CC, O’Donnell CP, O’Callaghan DJ, Lyng JG (2006). Dielectric properties of process cheese from 0.3 to 3GHz. Journal of Food Engineering.

[23] Metaxas, A. C. & Meredith, R. J. Industrial microwave heating. London: Peter Peregrinus Ltd (1983).

[24] Ohlsson, T. Dielectric properties and microwave processing in *Food properties and computer-aided engineering of food processing systems* (eds Singh, R. P. &Medina, A. G.) 73–92 (Springer Netherlands, 1989).

[25] Mudgett, R. E. Dielectric properties of food in *Microwaves in the food processing industry* (ed. Decareau, R. V.) (Academic Press, 1985).

[26] Nelson SO (2003). Frequency- and temperature-dependent permittivities of fresh fruits and vegetables from 0.01 to 1.8 GHz. Transactions of the ASAE.

[27] Wang Y, Zhang L, Gao M, Tang J, Wang S (2013). Temperature- and moisture-dependent dielectric properties of macadamia nut kernels. Food & Bioprocess Technology.

[28] Zhu X, Kang F (2015). Frequency- and temperature-dependent dielectric properties of goat’s milk adulterated with soy protein. Food & Bioprocess Technology.

[29] Bansal N, Dhaliwal AS, Mann KS (2015). Dielectric properties of corn flour from 0.2 to 10 GHz. Journal of Food Engineering.

[30] Trabelsi S (2015). Variation of the dielectric properties of chicken meat with frequency and temperature. Journal of Food Measurement and Characterization.

[31] Hu LZ, Toyoda K, Ihara I (2008). Dielectric properties of edible oils and fatty acids as a function of frequency, temperature, moisture and composition. Journal of Food Engineering.

[32] Martín-Esparza ME, Martínez-Navarrete N, Chiralt A, Fito P (2006). Dielectric behavior of apple (var. *Granny Smith*) at different moisture contents: Effect of vacuum impregnation. Journal of Food Engineering.

[33] Mudgett RE, Goldblith SA, Wang DIC, Westphal WB (1980). Dielectric behavior of a semi-solid food at low, intermediate and high moisture contents. Journal of Microwave Power.

[34] Guo W, Tiwari G, Tang J, Wang S (2008). Frequency, moisture and temperature-dependent dielectric properties of chickpea flour. Biosystems Engineering.

[35] Jiao S, Johnson JA, Tang J, Tiwari G, Wang S (2011). Dielectric properties of cowpea weevil, black-eyed peas and mung beans with respect to the development of radio frequency heat treatments. Biosystems Engineering.

[36] Li R, Zhang S, Kou XX, Ling B, Wang SJ (2017). Dielectric properties of almond kernels associated with radio frequency and microwave pasteurization. Scientific Reports.

[37] Zhu X, Guo W, Wang S (2014). Dielectric properties of ground hazelnuts at different frequencies, temperatures, and moisture contents. Transactions of the ASABE.

[38] Torrealbameléndez R, Sosamorales ME, Olveracervantes JL, Coronachávez A (2015). Dielectric properties of beans at different temperatures and moisture content in the microwave range. International Journal of Food Properties.

[39] Zhang S, Zhou L, Ling B, Wang S (2016). Dielectric properties of peanut kernels associated with microwave and radio frequency drying. Biosystems Engineering.

[40] Miller LA, Gordon J, Davis EA (1991). Dielectric and thermal transition properties of chemically modified starches during heating. Cereal Chemistry.

[41] Bosmans GM, Pareyt B, Delcour JA (2016). Non-additive response of blends of rice and potato starch during heating at intermediate water contents: a differential scanning calorimetry and proton nuclear magnetic resonance study. Food Chemistry.

[42] Leszczyński W (2004). Skrobia-surowiec przemysłowy budowa i właściwości. Zeszyty Problemowe Postępów Nauk Rolniczych.

[43] Jaska E (1971). Starch gelatinization as detected by proton magnetic resonance. Cereal Chemistry.

[44] Ryynänen S, Risman PO, Ohlsson T (1996). The dielectric properties of native starch solutions - a research note. Journal of Microwave Power and Electromagnetic Energy.

[45] Wang S (2003). Dielectric properties of fruits and insect pests as related to radio frequency and microwave treatments. Biosystems Engineering.

[46] Guo W, Wang S, Tiwari G, Johnson JA, Tang J (2010). Temperature and moisture dependent dielectric properties of legume flour associated with dielectric heating. LWT - Food Science and Technology.

[47] Goedeken DL, Tong CH, Virtanen AJ (1997). Dielectric properties of a pregelatinized bread system at 2450 MHz as a function of temperature, moisture, salt and specific volume. Journal of Food Science.

[48] Feng H, Tang J, Cavalieri RP (2002). Dielectric properties of dehydrated apples as affected by moisture and temperature. Transactions of the ASAE.

[49] Sosa-Morales ME (2009). Dielectric heating as a potential post-harvest treatment of disinfesting mangoes, part I: relation between dielectric properties and ripening. Biosystems Engineering.

[50] Guo W, Wu X, Zhu X, Wang S (2011). Temperature-dependent dielectric properties of chestnut and chestnut weevil from 10 to 4500 MHz. Biosystems Engineering.

[51] Bansal N, Dhaliwal AS, Mann KS (2016). Dielectric characterization of rapeseed (*Brassica napus L*.) from 10 to 3000 MHz. Biosystems Engineering.

[52] Torrealba-Meléndez R, Sosa-Morales ME, Olvera-Cervantes JL, Corona-Chávez A (2015). Dielectric properties of cereals at frequencies useful for processes with microwave heating. Journal of Food Science and Technology.

[53] Sharma GP, Prasad S (2002). Dielectric properties of garlic (*Allium sativum L*.) at 2450 MHz as function of temperature and moisture content. Journal of Food Engineering.

[54] Schiffmann, R. F. Microwave and dielectric drying. New York: Marcel Dekker (1995).

[55] Henry F, Costa LC, Serpelloni M (2003). Dielectric method for the determination of a_w_. Food Chemistry.

[56] Traffano-Schiffo MV, Castro-Giraldez M, Colom RJ, Fito PJ (2015). Study of the application of dielectric spectroscopy to predict the water activity of meat during drying process. Journal of Food Engineering.

[57] Guo W, Zhu X (2014). Dielectric properties of red pepper powder related to radiofrequency and microwave drying. Food & Bioprocess Technology.

